# Neural correlations between cognitive deficits and emotion regulation strategies: understanding emotion dysregulation in depression from the perspective of cognitive control and cognitive biases

**DOI:** 10.1093/psyrad/kkac014

**Published:** 2022-11-10

**Authors:** Wei Gao, XinYu Yan, JiaJin Yuan

**Affiliations:** The Affect Cognition and Regulation Laboratory (ACRLab), Institute of Brain and Psychological Science, Sichuan Normal University, Chengdu, Sichuan 610066, China; The Affect Cognition and Regulation Laboratory (ACRLab), Institute of Brain and Psychological Science, Sichuan Normal University, Chengdu, Sichuan 610066, China; The Affect Cognition and Regulation Laboratory (ACRLab), Institute of Brain and Psychological Science, Sichuan Normal University, Chengdu, Sichuan 610066, China

**Keywords:** depression, emotion dysregulation, emotion regulation strategies, cognitive control, cognitive biases

## Abstract

The link between cognitive function and emotion regulation may be helpful in better understanding the onset, maintenance, and treatment for depression. However, it remains unclear whether there are neural correlates between emotion dysregulation and cognitive deficits in depression. To address this question, we first review the neural representations of emotion dysregulation and cognitive deficits in depression (including deficits in cognitive control and cognitive biases). Based on the comparisons of neural representations of emotion dysregulation versus cognitive deficits, we propose an accessible and reasonable link between emotion dysregulation, cognitive control, and cognitive biases in depression. Specifically, cognitive control serves the whole process of emotion regulation, whereas cognitive biases are engaged in emotion regulation processes at different stages. Moreover, the abnormal implementation of different emotion regulation strategies in depression is consistently affected by cognitive control, which is involved in the dorsolateral, the dorsomedial prefrontal cortex, and the anterior cingulate cortex. Besides, the relationship between different emotion regulation strategies and cognitive biases in depression may be distinct: the orbitofrontal cortex contributes to the association between ineffective reappraisal and negative interpretation bias, while the subgenual prefrontal cortex and the posterior cingulate cortex underline the tendency of depressed individuals to ruminate and overly engage in self-referential bias. This review sheds light on the relationship between cognitive deficits and emotion dysregulation in depression and identifies directions in need of future attention.

## Introduction

Major depressive disorder (MDD), characterized by emotional dysfunction, is one of the most widespread mood disorders with a significant prevalence rate (Joormann & Stanton, 2016; Liu & Thompson, 2017). There has been an increase in depression prevalence around the world, which affects people throughout their lifetimes and causes significant financial, medical, and emotional strain on modern society (Otte *et al*., 2016; Richards, 2011). Further, depression is closely related to impaired cognitive function, reduced quality of life, and higher risk of suicide (Hansson, 2002; Hecimovic *et al*., 2012). Even worse, depression and anxiety have increased by 28.0 and 26.9%, respectively, since the COVID-19 pandemic (Gunnell *et al*., 2020; Hawes *et al*., 2021). Given the consequences of this epidemic in exacerbating depression, identifying the risk factors and underlying mechanisms of depression is crucial to providing effective prevention and therapeutic interventions. Many researchers have studied this to increase our understanding of the emergence, aggravation, and overcoming of depression.

Two principal potential mechanisms contribute to the development and maintenance of depression—emotion dysregulation and cognitive deficits (Paulus *et al*., 2016; Ravnkilde *et al*., 2002). Depression is closely linked to emotion dysregulation and individuals who tend to be depressed are likely to use maladaptive emotion regulation strategies (Liu & Thompson, 2017; Wante *et al*., 2017). Evidence of meta-analysis has indicated that depression is negatively correlated to the use of adaptive emotion regulation strategies (e.g. reappraisal, distraction) whereas it is positively correlated to the use of maladaptive emotion regulation strategies (e.g. rumination, suppression) (Schäfer *et al*., 2017). Moreover, recent studies have found people with difficulties in emotion regulation were more susceptible to depression and anxiety during the COVID-19 pandemic, which highlighted the importance of emotion regulation in maintaining psychological and physical health in a public emergency (Groarke *et al*., 2021; Low *et al*., 2021). On the other hand, a wide range of cognitive deficits has been consistently found in depression, including cognitive control (e.g. working memory, shifting, inhibition, and processing speed) (Kertz *et al*., 2016; Paulus, 2015) and cognitive biases (e.g. attention, interpretation, and self-referential processing) (Hallion & Ayelet Meron, 2011; Platt *et al*., 2017). Several studies have found that patients suffering from depression show a fixation on negative information and impaired general cognitive control (De Lissnyder *et al*., 2010; Joormann & Gotlib, 2008). For example, deficits in cognitive control hamper depressed people from getting over maladaptive responses (Joormann & Siemer, 2011). Further, several studies have indicated that the decreased well-being and emotional resilience during the COVID-19 pandemic were due to social isolation and a lack of activity, which impair cognitive function (De Pue *et al*., 2021; Halpern *et al*., 2020). Although both emotion dysregulation and cognitive deficits have proven to play critical roles in the emergence and aggravation of depression (Joormann & Tanovic, 2015; Villalobos *et al*., 2021), there is still a lack of systematic elaboration on the intrinsic neural correlations between them.

Clarifying this issue may be helpful in better understanding the mechanisms of depression and further improving treatment approaches (Fehlinger *et al*., 2013; Roiser *et al*., 2012). In this review, the main aim is to complement this gap in the field by providing a systematic overview of published studies on emotion dysregulation and cognitive deficits in depression. We will focus on evidence from the documentation of cognitive factors and emotion regulation in depression (Joormann & Stanton, 2016; LeMoult & Gotlib, 2019), instead of providing an elaborate inventory of depression studies. We begin with a brief review of the major evidence concerning emotion dysregulation and cognitive deficits in depression. We then review abnormal emotion regulation in the evidence for depression, and discuss important advances in the neural representation and correlation between emotion dysregulation and depression. Next, we discuss the major neural progress of cognitive biases in depression to clarify the association between it and cognitive deficits. Furthermore, we summarize the neuroimaging evidence on emotion dysregulation and cognitive deficits and try to link them, in particular, to help us gain insight into depression. Finally, we propose our insights into future directions of work that may deepen the knowledge of emotion dysregulation and cognitive deficits, which have crucial implications for improving depression.

## Emotion Dysregulation and Cognitive Deficits in Depression

Research on emotion regulation has indicated that emotional regulation is a complex process described as active or passive strategic processes that affect the generation, intensity, maintenance, and expression of emotional reactions (Campos *et al*., 2010; Gross & Barrett, 2011; Gross & Thompson, 2007). Despite the complexity of the definition, researchers generally agree that emotion regulation requires the involvement of different strategies to achieve the goal of resolving emotional problems (Aldao *et al*., 2010; McRae & Gross, 2020). Previous studies mostly focused on emotion regulation strategies, such as individual variability in the disposition and effectiveness of different strategies (John & Gross, 2004; Webb *et al*., 2012). Research on emotion regulation strategies has gradually been integrated into established cognitive models, which can help researchers to further clarify the deficits of depression (Joormann & Stanton, 2016; LeMoult & Gotlib, 2019). Moreover, numerous studies of depression have been devoted to examining whether depressed people have difficulties in implementing adaptive strategies and whether they are prone to the use of maladaptive emotion regulation strategies (Thompson *et al*., 2010; Wante *et al*., 2017).

Prior studies consistently indicated that dysfunctional patterns in emotion regulation have been identified as critical risk factors in depression. Specifically, several studies found ineffective implementation of adaptive emotion regulation strategies (e.g. reappraisal, distraction) in depression, which led to the failure of negative emotion remission (Liu & Thompson, 2017; Smoski *et al*., 2014). Cognitive reappraisal and distraction are two of the most common and frequently used strategies, and are considered as successful in reducing negative emotions. It has been shown that reappraisal and distraction are associated with a decreased incidence of depression, fewer negative effects, and increased subjective well-being (Birk & Bonanno, 2016; Efinger *et al*., 2019). Moreover, depression is also linked to the frequent use of maladaptive emotion regulation strategies, which are not considered to be conducive to alleviating negative emotions and lead to a vicious circle, such as cognitive inflexibility and failure to break free from negative emotions (e.g. rumination, expressive suppression) (Dryman & Heimberg, 2018; Spasojevic & Alloy, 2001; Whisman *et al*., 2020). Considering that multiple aspects of depression may be affected by adaptive and maladaptive strategies, in the later sections, we focus on these commonly mentioned strategies involved in depression: reappraisal, distraction, rumination, and suppression.

Researchers found that depression is associated with broad impairments in cognitive functioning, and cognitive deficits have been listed in the Diagnostic and Statistical Manual of Mental Disorders that can reflect depressive symptoms (Kendler, 2016; Martínez-Arán *et al*., 2004; Withall *et al*., 2009). However, what the pervasive cognitive deficits in depression are remains a controversial topic. The existing literature provides partial confirmatory evidence for the presence of cognitive control deficits in depression (Kircanski *et al*., 2012; Koster *et al*., 2017). For example, a recent meta-analysis found that depressed participants exhibited impairments in many aspects of cognitive control compared with healthy people, including updating, shifting, and inhibition (Dotson *et al*., 2020). More importantly, people relieving negative emotions under stressful conditions may benefit from higher cognitive control ability, which is believed to be related to depression (Gabrys *et al*., 2018; Grahek *et al*., 2019). Specifically, some previous studies found that low levels of reappraisal and distraction in stressful situations were associated with decreased cognitive control (Sandner *et al*., 2021). In addition, individual differences in cognitive biases (e.g. attention, interpretation, and self-referential processing) can also influence one's capacity for emotion regulation; these biases provide the potential possibility for and vulnerability to depression (Hallion & Ayelet Meron, 2011; Platt *et al*., 2017). For instance, it has been uncovered in research on attention bias that negative attention bias is related to emotional responses to stress or emotional induction (Macatee *et al*., 2017; MacLeod *et al*., 2002). Indeed, previous researchers proposed that abnormal function in cognitive control and cognitive biases may contribute to dysfunctional implementation of adaptive and maladaptive emotion regulation strategies (Joormann & Quinn, 2014; Yoon & Rottenberg, 2020). For more details, we recommend readers to look at the recent reviews (Joormann & Stanton, 2016). Therefore, here we are mainly concerned about the cognitive bias and cognitive control, in particular, highlighting the neural correlates of these cognitive deficits to emotion regulation. As well, we want to discuss the cognitive factors that may occur at different stages of emotion generation and serve as specific emotion regulation strategies.

## Neural Representations of Emotion Regulation Strategies in Depression

### Adaptive emotion regulation strategies

#### Cognitive reappraisal

Cognitive reappraisal is interpreted as the attempt to modify individuals’ emotional experience by reinterpreting affective stimuli or events in a way that involves changing thoughts and beliefs, and has been generally regarded as an adaptive emotion regulation strategy (McRae *et al*., 2012a; Wallace-Hadrill & Kamboj, 2016). Previous experimental studies found reappraisal can help people reduce negative emotional experiences and physiological arousal in nonclinical samples (Mauss *et al*., 2007; Ray *et al*., 2010). Similarly, several clinical studies showed that less frequent habitual and effective use of reappraisal has been linked to higher depression severity (Fladung *et al*., 2010; Shapero *et al*., 2019). Moreover, imaging evidence of reappraisal is multifarious, and indicates that reappraisal mainly involves hyperactivity in semantic and perceptual areas of the lateral temporal cortex and deactivation in the amygdala and insula, which is accompanied by increased activation in prefrontal and parietal brain areas (Smoski *et al*., 2013; Zilverstand *et al*., 2017).

Functional neuroimaging evidence consistently found a robust association between reappraisal and prefrontal and subcortical regions in MDD (Johnstone *et al*., 2007; Perchtold *et al*., 2017). Specifically, individuals with depression showed dysfunction in the dorsomedial (dmPFC), the left dorsolateral (dlPFC) and the ventrolateral prefrontal cortex (vlPFC), and failed to reduce hyperactivity in subcortical regions (e.g. amygdala and insula). For example, prior research found that depressed adolescents engage the left dmPFC and the left dlPFC less efficiently and have greater amygdala activation than healthy controls, which reduces reappraisal success (LeWinn *et al*., 2018). Another study of reappraisal also revealed that successful emotion regulation relied on the engagement of dmPFC as well as dlPFC and vlPFC in both high- and low-intensity emotional conditions (Silvers *et al*., 2015). Moreover, a recent study indicated that the link between habitual cognitive reappraisal and depressive symptoms is affected by the microstructural circuit of the pathway of the amygdala and prefrontal cortex (d'Arbeloff *et al*., 2018). The neural integration model by Ochsner and Gross provided a major interpretation for these studies (Ochsner & Gross, 2005). They proposed two different cognitive control top-down systems: the dmPFC and the dlPFC help individuals to revalue emotional information, and the vlPFC is involved in learning the link between emotion-related outcomes and emotional contexts. The main role of these prefrontal brain regions is to help individuals modulate subcortical areas during reappraisal.

Additionally, MDD studies of different reappraisal types also found several special activated brain regions. Specifically, several studies have suggested that the implementation of voluntary reappraisal processes also activated the parietal and temporal regions (Belden *et al*., 2014; Wang *et al*., 2017), while the implementation of automatic reappraisal processes showed the activation of the anterior cingulate cortex (ACC) and the orbitofrontal cortex (OFC) (Kanske *et al*., 2012; Munoz *et al*., 2014). For instance, Hua and colleagues discovered that individuals who have reduced neural reactions in the left inferior parietal area during the reappraisal task exhibit emotional instability and worse depressive symptoms in daily life (Hua *et al*., 2020). Moreover, a previous study proposed that depressed individuals completed successful reappraisal by excessively recruiting the medial prefrontal cortex and the ACC in the early stages of the automatic emotion regulation process (Rive *et al*., 2013). Several recent studies of spontaneous reappraisal indicated that the modulation of amygdala activation by the OFC may be essential for alleviating negative emotions (Gao *et al*., 2021; Khawli *et al*., 2017). Besides, another previous study suggests that the link between reappraisal and depression was affected by the temporal dynamics of the medial prefrontal regions, specifically the orbitofrontal area (Gao *et al*., 2018). In sum, though the existing evidence is mixed, it is almost certain that individuals with depression mainly involve the frontal, parietal, and temporal cortices during reappraisal, which is associated with the activity of subcortical regions.

#### Distraction

Another adaptive coping with depression is generally regarded as distraction, which is conceptualized as an attentional emotion regulation strategy. Early theoretical studies proposed that distraction is an effective emotion regulation process by using sufficient cognitive resources to stop individual negative emotional thoughts (Sheppes *et al*., 2014). Researchers also found that distraction may be an effective strategy in clinical samples, individuals with depression reported they believe that distraction would likely help alleviate their negative mood (Teismann *et al*., 2012). For example, previous studies found that individuals with depression can successfully use distraction to improve negative feelings via being instructed. However, depressed patients show less frequency and possibility of using distraction than healthy people; they refuse to be distracted because it interrupts them from focusing on themselves and understanding personal problems (Joormann & Vanderlind, 2014). Moreover, recent studies suggested that distraction is limited to short-term gains and has no durable gains and adaptive capacity (Sheppes & Gross, 2011; Sheppes *et al*., 2014). These reasons lead to scattered and incomplete imaging evidence about the relations between distraction and depression, hampering researchers from drawing convergent conclusions. Nevertheless, several comparative studies on reappraisal have provided potential evidence to understand neural representations of distraction in depression.

The brain regions involved in reappraisal and distraction show different and similar components, implying that different subprocesses of the emotion regulation model may be controlled by overlapping neural regions, as well as retain partial neural specificity of each subprocess (Kanske *et al*., 2010; McRae *et al*., 2010). First, similar to reappraisal, previous studies have also examined distraction (i.e. distracting oneself by thinking about irrelevant or positive things) by using laboratory experiments, identifying the general patterns of prefrontal and subcortical regions' activation during distraction. Both reappraisal and distraction downregulate depressed individuals’ amygdala activity by engaging a common network of control areas, including dmPFC, dlPFC, and dorsal anterior cingulate cortex (dACC). Previous studies showed the efficiency of distraction in alleviating subjective negative emotional feelings and amygdala activity. For example, Kanske and colleagues found that left dmPFC activity was increased in MDD participants relative to healthy people during distraction (Kanske *et al*., 2012). Other enhanced activities of prefrontal regions (e.g. dmPFC and vlPFC, which included the dACC) have been found in MDD participants relative to healthy people during automatic attentional shifting (Bettis *et al*., 2021; McRae *et al*., 2010). Second, relative to reappraisal, depressed individuals may exhibit stronger activations in vlPFC and bilateral superior parietal cortex during distraction. Moreover, a recent study suggested that these two cortical regions showed relative functional specificity for distraction (vlPFC) and reappraisal (dlPFC) strategies in downregulating socially relevant emotion (Zhao *et al*., 2021). Additionally, another previous study found that the bilateral superior parietal cortex exhibited a large range of neural activity in the distraction condition, where these brain regions overlapped but showed stronger responses to the activation for reappraisal (Kanske *et al*., 2010). Finally, distraction showed a unique activation in the inferior parietal cortex (IPC) and postcentral gyrus, which possibly reflects the process of attentional disengagement required to shift attention away from negative contexts (Dörfel *et al*., 2014). IPC activations might affect attentional orienting and shifting processes to guide attention away from the perceived negative stimulus toward other irrelevant things during distraction.

To summarize, although imaging neuroscience evidence about distraction and depression is mixed and insufficient so far, the literature suggests that depression may be related to less frequent and inefficient use of adaptive strategies (distraction and reappraisal). Given that the neural findings of distraction have not been fully clarified, researchers have proposed that distraction may not be adaptive for long-term coping with depression and these comments should be considered in future studies of emotion regulation. Even so, limited research evidence suggests both common (dmPFC, dlPFC, and dACC) and distinct (vlPFC and IPC) patterns of prefrontal activation during distraction compared to reappraisal.

### Maladaptive emotion regulation strategies

#### Rumination

Rumination is generally considered to be maladaptive and has been defined as a cognitive pattern characterized by a rigid and persistent negative thought, particularly an irresistible concern for self-relevant information (Koval *et al*., 2012; Nolen-Hoeksema *et al*., 2008). Plenty of evidence supported the idea that higher rumination levels were associated with the exacerbation and maintenance of mood-related diseases, particularly with depressive symptoms (Hamlat *et al*., 2015; Liverant *et al*., 2010). Accumulating neuroimaging evidence from empirical studies has emphasized the association between rumination and cognitive control deficits, as well as self-referential processing and recall of autobiographical memories (Fawcett *et al*., 2015; Hamlat *et al*., 2015). To date, brain areas of the default mode network (DMN) and subgenual prefrontal cortex have been found and considered the most consistently neurologically relevant regions in studies of rumination and depression (Hamilton *et al*., 2015; Murphy *et al*., 2016). Understanding the function of these brain regions involved in rumination may help illuminate critical risk factors implicated in depression.

Previous studies of depression have found links between altered neural activity in the inferior frontal gyrus, anterior cingulate cortex, inferior parietal lobe, and medial frontal gyrus primarily involved in task-related control networks, which are associated with the rumination process. These brain regions mainly comprise the lateral prefrontal cortex and parietal cortex, which may be correlated with cognitive modulation during the inhibition process (Kucyi *et al*., 2014; Kühn *et al*., 2012). For example, a previous study reported that lower dlPFC activity is correlated with higher rumination when depressed individuals were asked to ignore negative stimuli in emotional situations to complete the cognitive task (Siegle *et al*., 2002). Moreover, several studies found decreased mPFC activity in depressed individuals during ruminating compared to healthy people, suggesting decreased top-down inhibition processes (Mandell *et al*., 2014; Nejad *et al*., 2019). The relevant results are further reinforced by findings of altered activity within the DMN during rumination. Previous studies have shown that mPFC, posterior cingulate cortex (PCC), and medial temporal gyrus (MTG) displayed higher activation in adults with MDD during rumination (Burkhouse *et al*., 2017; Zhou *et al*., 2020). As such, rumination has been linked to brain regions for cognitive control, which provides a critical substrate for understanding depression and may shed some light on the pathogenesis of depression.

In addition, the association between rumination and abnormal self-referential processing in depressed individuals may be reflected by some specific brain regions including PCC, subgenual prefrontal cortex (sgPFC), and ventromedial prefrontal cortex (vmPFC) (Perkins *et al*., 2015; Zhu *et al*., 2017). For instance, a meta-analysis of depressive rumination found that the extent of individual rumination can be predicted by the functional connectivity of the sgPFC and DMN (Makovac *et al*., 2020; Zhou *et al*., 2020). Several studies also indicated that individuals with pathological rumination have a self-focused tendency and negative autobiographical memory, which is related to abnormal activations of the PCC in depression. The sgPFC and PCC were also involved in the processes of recalling one's own experiences and reflecting on themselves, which may moderate self-referential memory during rumination (Jacob *et al*., 2020; Zhou *et al*., 2020). Moreover, Lemogne and colleagues provided indirect imaging evidence that vmPFC activity plays a role in the self-focusing rumination process, which is associated with the development of acute depression and a higher risk of relapse (Lemogne *et al*., 2012). The vmPFC may facilitate self-referential processing by representing visualization or extracting similar situational content to give value to objectives. Furthermore, our recent work suggested that decreased temporal variability in the vmPFC region may implicate cognitive inflexibility and persistent immersion in self-referential memories, which lead to a higher rumination and depressive risk (Gao *et al*., 2022). As mentioned before, previous neuroimaging findings of depression implied that rumination is generally related to abnormal activations of the lateral prefrontal cortex and parietal cortex, and specifically these core regions of the DMN (e.g. sgPFC, PCC, and vmPFC). These previous findings provide the basis and insight for understanding the rumination process in depression.

#### Expressive suppression

Expressive suppression has considered an emotion regulation strategy in which individuals try to restrain the consequences of emotional information on the inner (e.g. physiological) and outer (e.g. emotional expression) states (Cutuli, 2014). As a response-focused strategy, it often fails to decrease the emotional experience as this regulation process often takes effect after full emotion generation (Paul *et al*., 2013). Although a few studies indicated that expressive suppression has cultural differences in effectiveness and may help reduce acute negative effects by providing instructions among depressed participants, other findings have reported that suppression is correlated to increased negative affect, and lower well-being and relationship satisfaction (Cutuli, 2014; Soto *et al*., 2011). The evidence from clinical samples also indicated that individuals who suffered from depression had a high frequency of using suppression (Dryman & Heimberg, 2018; Larsen *et al*., 2013). Besides, neuroimaging studies on habitual use of suppression in depression have found that this strategy is not only ineffective in reducing negative emotional experiences, but also adds the cognitive burden and more severe emotional consequences (Butler *et al*., 2003; Cutuli, 2014).

Few studies directly explored the neural basis of expressive suppression processes in depressed patients. However, several functional neuroimaging studies provide supplementary evidence by manipulating different emotion regulation strategies (expressive suppression versus cognitive reappraisal) (Goldin *et al*., 2008; Vanderhasselt *et al*., 2013). In an early review, Phillips and colleagues found that expressive suppression was correlated with brain activities in the left vlPFC, right dlPFC, and bilateral dmPFC (Phillips *et al*., 2008). However, other functional magnetic resonance imaging (fMRI) studies did not consistently report modulation of activation in prefrontal regions during expressive suppression (Berboth & Morawetz, 2021; Dörfel *et al*., 2014). A study of brain structure showed that the larger the gray matter volume of the dmPFC, the greater tendency individuals use expressive suppression (Hermann *et al*., 2014). Researchers also found that successful suppression of negative feelings involved coactivation of the dACC and dmPFC when pariticpants performed a voluntary suppression task (Lévesque et al., 2003).Moreover, a recent study examined longitudinal correlations between emotion regulation and depression severity in adolescents; their results showed that diminished prefrontal cortical function, in particular decreased activities of the dmPFC, affect the use of expressive suppression (Vilgis *et al*., 2018). Additionally, neuroimaging studies in clinical samples found that both cortical and subcortical regions were associated with expressive suppression. The subcortical regions mainly included the amygdala and hippocampus which were related to emotion generation and memory, while the cortical regions mainly included the dmPFC, dlPFC, and dACC, which are generally associated with the function of self-control, regulating emotion-expressive behavior, and voluntary inhibition on the action (Giuliani *et al*., 2011; Sikka *et al*., 2022).

In addition, except for these emotional control regions, expressive suppression is also associated with some particular cortical and subcortical regions compared with cognitive reappraisal. Recent studies have found that the supplementary motor area (SMA) and the superior frontal sulcus were more engaged in voluntary suppression tasks, which suggested that these regions may be critical locations for inhibiting the expression of emotional responses (Picó-Pérez *et al*., 2017). In another fMRI study, Giuliani and colleagues observed the relationship between the volume of the anterior insula and different strategies, and found a positive link with expressive suppression but not with cognitive reappraisal (Giuliani *et al*., 2011). Moreover, previous studies on depression underline that abnormal activation in the right dlPFC may interrupt the top-down control and inhibition processes during expressive suppression, which leads to increases in subcortical activation (Vrticka *et al*., 2013). Similarly, another study about suppression strategy suggests that hyperactive right prefrontal cortex(e.g. right ventrolateral prefrontal cortex), which is implicated in overactive inhibitory function, may disrupt the ability of MDD patients to experience positive emotions in the condition of downregulating positive emotions (Light *et al*., 2011; Rive *et al*., 2013). These studies of expressive suppression are mixed and inconsistent since direct research is insufficient, it is necessary to conduct independent and systematic studies about expressive suppression in the future study of depression.

## Neural Representations of Cognitive Deficits in Depression

### Cognitive control

Cognitive control is recognized as a top-down process that supports flexible responses and consists primarily of goal-oriented processes such as goal modification (updating or maintenance), response modulation (inhibition or suppression), and online monitoring, all of which are important components of adaptive self-regulation (Gratton *et al*., 2018; Pruessner *et al*., 2020). These processes are disrupted in many psychiatric disorders including depression, which highlights cognitive control dysfunction as a possible pathogenic risk factor for mood disorders (Fales *et al*., 2008; Joormann & Tanovic, 2015). Numerous previous studies demonstrated that depressed individuals showed symptoms of difficulties detaching from, and suppressing, the processing of negative emotional information, which are closely associated with cognitive control dysfunctions (Grahek *et al*., 2018; Kircanski *et al*., 2012). A recent systematic overview indicated that both major and subthreshold depression patients have a close relation with deficits in cognitive control, and the relationship was persisting throughout the whole lifespan (Dotson *et al*., 2020). Given the significant predicting effect of cognitive control dysfunctions on depressive symptoms, neuroimaging techniques have been used to explore the relationship between them.

Multiple previous studies indicated that changes in the activity of the dlPFC were linked to diminished cognitive control ability in depressed individuals (Fales *et al*., 2008; Joormann & Vanderlind, 2014). For example, researchers have found decreased metabolic activity in the dlPFC in depressed individuals using fMRI and positron emission tomography (Ko *et al*., 2013). Similarly, clinical sample studies also found a significant association between sustained amygdala reactivity and lower dlPFC activity during emotional and cognitive tasks, respectively (Siegle *et al*., 2007). Consistent with these findings, depressed elderly patients also showed hypoactivity of the dlPFC and low functional connectivity between the dACC and dlPFC in cognitive conflict tasks (Aizenstein *et al*., 2009; Alexopoulos *et al*., 2012). Moreover, a neurological intervention study of depression indicated that anodal transcranial direct current stimulation (tDCS) applied to the left dlPFC improves deficient cognitive control in MDD (Wolkenstein & Plewnia, 2013). This neuroimaging evidence highlight that the top-down regulation of cognitive processing can benefit from the dlPFC activities, which also play a critical role in dealing with emotions.

Other crucial brain areas engaged in cognitive processes like the ACC and the dmPFC have also been widely mentioned in depression studies, which mainly come from the evidence of automatic cognitive control studies (Kanske *et al*., 2012; Wagner *et al*., 2008). In previous studies mainly concerned with the input and processing of emotional materials, researchers found the abnormal response of the dmPFC and the ACC and other areas were closely associated with cognitive control in emotional tasks (Ochsner *et al*., 2012; Rive *et al*., 2013). Moreover, a recent review also pointed to the important role of the activation and connectivity of the ACC in depressed individuals during emotional tasks requiring sufficient cognitive control (Grahek *et al*., 2018). Abnormal activation of the ACC may be associated with blunted feelings of emotional awareness, diminished capacity to respond in an uncertain context, and conflict between an individual's emotional state and external information (Alexopoulos *et al*., 2012; Rive *et al*., 2013). In addition, studies of brain structural and functional networks have identified microstructural white matter abnormalities in the dlPFC and the ACC in depressed adults during cognitive control and emotional regulation (Alexopoulos *et al*., 2009; Wang *et al*., 2016). This evidence suggests that depression is mainly correlated with the impaired function of the dlPFC and the ACC during the regulation of affective information.

### Cognitive biases

According to theoretical models of depression, negative schemas can affect one's perception of self-relevant information and develop cognitive biases, including biases in attention, memory, and interpretation (Disner *et al*., 2011). Neuroimaging studies over the last decades have extended evidence about these cognitive biases in depression; these findings are mostly focused on aspects of interpretation, attention, and self-referential processing (Platt *et al*., 2017). First, to date, despite existing mixed results of negative interpretation bias in depression, there is growing evidence that individuals with depression prefer to interpret ambiguous content negatively (Hindash & Amir, 2012). For example, prior studies of adult and adolescent depression have found negative biases in interpreting both social and nonsocial information (Everaert, Podina, *et al*., 2017; Orchard *et al*., 2016). Researchers also found that depressive symptom severity has been associated with interpretation biases and have proposed that interpretation biases as vulnerability factors for future depressive episodes (Hindash & Amir, 2012; Normansell & Wisco, 2017). Evidence from cognitive neuroscience indicated that the vmPFC regions, particularly the OFC, play an important role in negative interpretation biases (Papageorgiou *et al*., 2017; Volz & von Cramon, 2009). Recent studies found the modulation of the interpretation bias is associated with changes in neural activities of the vmPFC (including OFC) in depressed individuals (Nejati *et al*., 2021; Nejati *et al*., 2022). Moreover, a study of interpretation bias modification indicated that increased neural activities of the bilateral OFC were correlated with relieved anxious symptoms and altered neural reactions to pleasure and anger feelings (Stoddard *et al*., 2016). These findings suggest the function of the OFC may contribute to interpretation bias.

Second, in the studies of attentional bias, researchers used the dot-probe task and eye tracking to examine the method of processing negative information in depressed individuals (Torrence & Troup, 2018; Waechter *et al*., 2014). A growing number of studies show that those individuals with depression have difficulty separating their attention from negative emotional information, which is associated with adverse responses in high-stress situations. For instance, compared to healthy control, depressed participants not only have significantly longer average glance durations for pictures featuring sadness and loss, but also spend less time looking at positive pictures (Eizenman *et al*., 2003; Joormann & Stanton, 2016). Evidence from electrophysiology indicated that the frontal and parietal regions are associated with attention bias (Grimshaw *et al*., 2014). Similarly, several fMRI studies also found abnormal activities in the parietal and prefrontal areas, particularly in the vlPFC and IPC (Bareham *et al*., 2018; Passarotti *et al*., 2010). For example, Chen and colleagues found abnormalities in the IPC may lead to attentional deficits and disturbing decision-making, enhancing suicide risk in depressive patients (Chen *et al*., 2015). Besides, Dedovic and colleagues observed subclinical individuals with depression have a positive correlation between attentional bias and left IPC and right vlPFC (Dedovic *et al*., 2014). These regions are a portion of the top-down attentional control network and have previously been found to engage in the process of modulating attention bias toward emotional stimuli.

Finally, emotionally consistent memory biases, such as preferential retrieval and automatic processing of negative information, are thought to reflect the individual's potential negative memory patterns (Del Valle & Mateos, 2018; Disner *et al*., 2011). Recent studies have shown that the emergence and aggravation of depression are closely related to over activity of the DMN, which is primarily involved in autobiographical memory and internal states of self-referential processing (Gao *et al*., 2022; Sheline *et al*., 2009). Increasing evidence from adult, adolescent, and child samples showed that depressed individuals have more negative self-referential memory biases than healthy people. For example, Auerbach and colleagues found that depressed individuals were more likely to endorse self-descriptive negative words than positive words (Auerbach *et al*., 2016). Convergent findings of neurological evidence indicated that a core pair of the DMN hubs, the PCC and the sgPFC, facilitate self-referential processing, which exhibit abnormal activation in depressed individuals. Previous studies found that the sgPFC displayed an over-activated response in healthy and depressed individuals during grief and negative self-reflection tasks (Hamilton *et al*., 2015). Moreover, compared to healthy individuals, researchers found enhanced functional couplings between the PCC and the sgPFC in MDD, which reflects a state of excessive negative self-focus and negative affective memory processing (Rosenbaum *et al*., 2017). Additionally, in a meta-analytic work on depression, a proposed model suggests that the abnormal integration between the DMN (particularly the PCC) and the sgPFC is closely correlated to depression (Zhou *et al*., 2020). Taken together, the activity and connection of the PCC and the sgPFC may contribute to the negative biased self-referential processing, which reflects the existence of negative memory patterns in depression.

## Neural Correlations between Emotion Dysregulation and Cognitive Deficits

So far, although the results of depression studies are still mixed, the neuroimaging evidence presented here provides a deducible conclusion: the effects of cognitive control on different emotion regulation strategies are consistent and universal, while the relationship between cognitive biases and different emotion regulation strategies may be distinct and specific. On the one hand, the key brain regions (such as the dlPFC, the dmPFC, and the ACC) that dominate cognitive control are consistently involved in depression studies about the abnormal use of emotion regulation strategies. For example, researchers found the dlPFC is not only the vital brain region that contributes to cognitive control but is also beneficial to the implementation of different emotion regulation strategies, including more persistent use of maladaptive strategies as well as less use of and difficulties in effectively implementing adaptive strategies (Joormann & Stanton, 2016; Joormann & Vanderlind, 2014). Similarly, in the process of neural development and maturation, the close association of the prefrontal brain regions between cognitive control and emotion regulation strategies is also demonstrated (Bunge & Crone, 2009; Crone & Steinbeis, 2017). Moreover, convergent evidence showed that decreased cognitive control and emotion dysregulation frequently occur together in depressed individuals, which may be attributed to abnormal activities in the dlPFC, the dmPFC, and the ACC (Banks *et al*., 2007; Berboth & Morawetz, 2021). Besides, neuroimaging evidence from developmental psychology indicated that interactions between the ability of cognitive control and emotion regulation during the structure and function of the prefrontal cortex becomes complete and mature in children (Mcrae *et al*., 2012b). Therefore, we thought the association of cognitive control with emotion regulation is stable and universal rather than specific to different emotion regulation strategies. It is important to understand the functions of the dlPFC, the dmPFC, and the ACC for improving the ability of cognitive control and emotion regulation together.

On the other side, the relationship between cognitive biases and different emotion regulation strategies may be distinct and specific. First, negative interpretation biases undermine the capability to reconstruct contextual information and disrupt access to adaptive strategies (e.g. reappraisal). As mentioned before, the OFC considered as a higher-level cortical structure and assigns value to stimuli in emotional situations: it is generally involved in depressed individuals with interpretation biases and ineffective reappraisal (Kanske *et al*., 2015; Wallis & Rich, 2011). For example, previous studies found that healthy individuals operate successful reappraisal of negative stimuli with activation of the OFC, which decreases activity in the amygdala and results in an attenuated emotional response (Gao *et al*., 2021; Roelofs *et al*., 2009). Abnormal activities of the OFC in individuals with depression during reappraisal may lead to biases in interpretation and make it difficult to use reappraisal, and cause inflexible and inappropriate responses (Siemer & Reisenzein, 2007). The overlapping neural evidence of the OFC in interpretation biases and reappraisal suggests the close association between interpretation biases and ineffective reappraisal in depressed individuals.

Second, attentional biases may lead depressed patients to difficulties in extricating themselves from affective loops, discouraging them from using effective emotion regulation strategies. Previous evidence suggested the vlPFC and the IPC in depressed individuals have distinctive activation patterns of the prefrontal cortex during distraction compared to reappraisal (Kanske *et al*., 2010; McRae *et al*., 2010). The vlPFC is mainly in charge of the attentional control processes related to stimulus priming and is considered to be a subfunctional area of the ventral attentional network; the IPC acts on attentional modulation processes through a top-down control system and is subordinate to the dorsal attentional network. Consistent with these functional associations, studies of depressed individuals with attentional biases point out that the vlPFC and the IPC involvement in disengaging from negative stimuli, which play an important role in the use of distraction (Bareham *et al*., 2018; Passarotti *et al*., 2010). Moreover, studies on attentional bias training also showed that individuals can use distraction to attend to looked less at negative images during a stressful situation (Baert *et al*., 2010; Lichtenstein-Vidne *et al*., 2017). These findings provide the most direct support for the idea that individual differences in attentional biases may lead to changes in the use of distraction.

Third, the PCC and the sgPFC have been considered two important regions involved in rumination and self-referential processing. Substantial evidence suggests that the processes of seeking and extracting information from autobiographical memory are linked to over-recruitment of the PCC regions (Leech & Sharp, 2014). Several studies have also shown that pathological rumination and attentional self-focusing in depression is governed by the activity pattern of PCC (Cheng *et al*., 2018; Granados-Domínguez *et al*., 2013). The abnormal activity in the sgPFC may reflect a maladaptive pattern of self-referential thought with rumination that is associated with a heightened risk of depression and other mental illnesses. For instance, previous researchers argue that a core aspect of maladaptive rumination was affected by hyper-connectivity of the DMN and sgPFC, which leads to a pattern of hyper-negative self-concern (Bratman *et al*., 2015; Hamilton *et al*., 2015). Moreover, one study of electroencephalography found that increased functional connectivity between the PCC and the sgPFC was associated with maladaptive rumination; this may be pivotal in generating self-referential cognitions with depression (Benschop *et al*., 2021). Altogether, these findings suggested that the PCC and the sgPFC might underlie the trend toward rumination and excessive involvement in self-referential thinking in depressed individuals.

Finally, no consistent and specific evidence were found in cognitive biases and suppression. Evidence from suppression studies showed that abnormal activities of brain regions such as the dlPFC, the dmPFC, and the ACC are generally associated with the functions of self-control, regulating emotion-expressive behavior, and voluntary inhibition of action, which are commonly involved in cognitive control (Giuliani *et al*., 2011; Sikka *et al*., 2022). Previous researchers argue that cognitive biases occur mostly before the emotional response is fully developed, while the influence of cognitive control runs through the whole process of emotion generation (Joormann & Stanton, 2016; LeMoult & Gotlib, 2019). Therefore, it is not surprising and plausible that suppression has no particular relation with cognitive biases but is generally influenced by cognitive control because suppression is a response-focused strategy, which decreases negative feelings through suppressing behavioral performance and typically appears after emotional responses have already arisen (Dryman & Heimberg, 2018; Paul *et al*., 2013).

**Figure 1:**
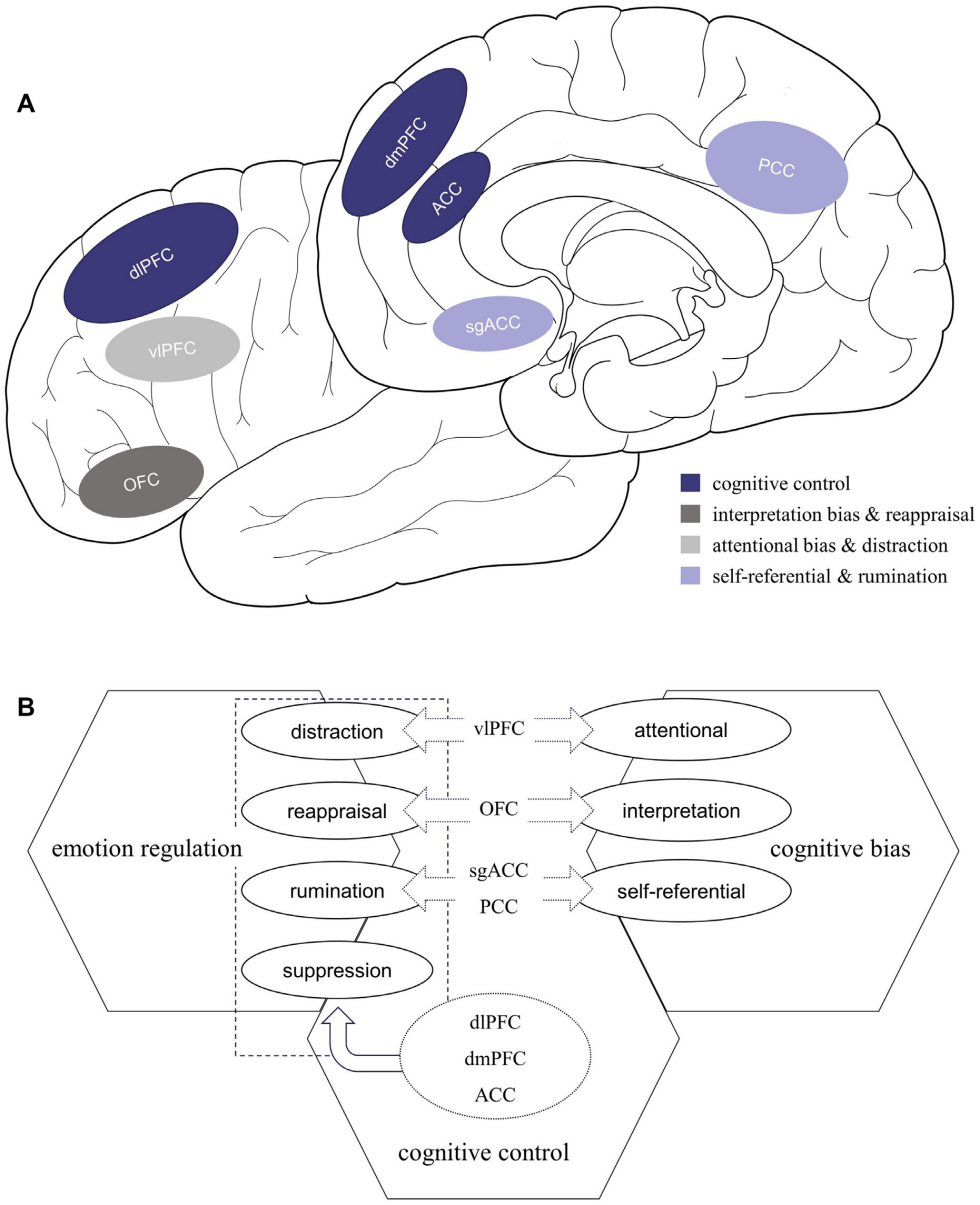
(A) The neural correlations between cognitive deficits and emotion regulation strategies. (B) The interconnection model between emotion regulation and cognitive deficits.

In sum, although the evidence is limited and unproven, the current inference is reasonable that the effects of cognitive control on different emotion regulation strategies are consistent and universal, while the relationship between cognitive biases and different emotion regulation strategies may be distinct (Fig. 1). Specifically, cognitive control is involved in the dlPFC, the dmPFC, and the ACC, which run through the whole process of emotion generation. Moreover, the OFC have been associated with interpretation bias and the vlPFC is associated with attentional bias, which contributes to the processes of reappraisal and distraction, respectively. Besides, the sgPFC and the PCC are engaged in self-referential bias underlying the process of rumination. Understanding the association of cognitive control and cognitive biases with different emotion regulation strategies can contribute to future research on mechanisms underlying the emergence, aggravation, and overcoming of depression.

## Limitation and Future Directions

Emotion dysregulation and cognitive deficits are the hallmark features of depression, which reflect a failure in self-regulation including difficulties implementing effective strategies and the use of ineffective strategies (Paulus *et al*., 2016; Ravnkilde *et al*., 2002). In recent decades, researchers have explored the neural mechanism of emotion dysregulation and cognitive deficits in depression, and have achieved many interesting but complicated findings. Our review of the literature provides a traceable and accessible link between emotion dysregulation and cognitive deficits by sorting through the neuroimaging evidence on different emotion regulation strategies. This review opens novel avenues for future research that explore the link between emotion dysregulation and cognitive deficits in depressed patients based on different emotion regulation strategies.

First, there is a need to directly examine the relationship between different emotion regulation strategies and cognitive control and cognitive bias based on neuroimaging, as the available research evidence is still scarce and mixed. For example, neuroimaging evidence of distraction and suppression is mostly contained in studies of reappraisal and rumination (Hermann *et al*., 2014; Kanske *et al*., 2015), which prevented researchers from clarifying the neural association between distraction and suppression and cognitive deficits. In addition, we cannot exclude the interaction between cognitive deficits and emotion regulation, it is necessary to directly examine the effect of cognitive deficits on emotion regulation in future studies.

Second, as the relationship between the two concepts of cognitive control and cognitive bias has not yet been clearly understood, the cognitive components of both and their role in the process of emotion regulation need to be further defined and tested. Some studies emphasize the effect of cognitive control on cognitive bias, arguing that cognitive control is a more fundamental cognitive process than cognitive bias (Everaert, Grahek, *et al*., 2017; LeMoult & Gotlib, 2019), while other studies argue that cognitive control and cognitive bias are two parallel processes with mutual influence that differ in the time course of cognitive processing (Joormann & Vanderlind, 2014; Villalobos *et al*., 2021). It is necessary to further refine and distinguish the association between cognitive control and cognitive bias in future studies because both of them have important implications for emotion regulation.

Third, there is growing evidence indicating that adaptation and maladaptation of emotion regulation strategies may be related to situational factors. For example, previous studies found that cognitive reappraisal, which is considered adaptive, may be inappropriate in high-arousal situations (Silvers *et al*., 2015; Troy *et al*., 2013); whereas expression suppression, which is considered maladaptive, can successfully regulate individual negative emotions in stressful situations (Roos *et al*., 2018; Yuan *et al*., 2014). Recently, researchers have begun to underline the importance of context, emotion regulation flexibility, and adaptiveness (Aldao *et al*., 2015; Pruessner *et al*., 2020). Given that depression is typically characterized by cognitive rigidity, these directions of research may be valuable in understanding the intrinsic mechanism of depression.

Fourth, another important feature of depression is anhedonia (Jordan *et al*., 2018; Treadway & Zald, 2011), however, most of the current evidence comes from studies on negative emotion downregulation, which may be insufficient for researchers to gain a comprehensive understanding of depression. Future research should focus more on the response to positive emotions in depression, as well as the use of emotion regulation strategies and cognitive processes in positive situations.

Finally, previous studies have developed treatments and cognitive training for depression based on improving emotion dysregulation and cognitive deficits, but most of these studies tend to focus on one specific aspect. For example, some recent studies have conducted cognitive bias modification and cognitive control training to reduce depressive symptoms in MDD (Koster & Hoorelbeke, 2015; Koster *et al*., 2017). Other studies have attempted to intervene in depression by adapting the use of different emotion regulation strategies (Berking *et al*., 2013; Mennin & Fresco, 2014). The current review suggests the broad influence of cognitive control on emotion regulation and the unique association between different types of cognitive biases and different emotion regulation strategies. Future research should try to develop cognitive bias modification training methods that are specific to different emotion regulation strategies, and can be combined with cognitive control training that yields sufficient available cognitive resources to improve top-down control of negative emotion.

In sum, we have provided several advances in understanding the relationship and role of cognitive deficits and emotion dysregulation in depression. We believe that this review will be critical to further integrating the emotion regulation and cognitive model of depression. In the future, it is necessary and promising to explore how cognitive deficits promote or hamper emotion regulation, to identify risk factors, and improve approaches to therapy for depression.
